# A Pediatric Case of Vertebral Osteomyelitis Caused by Aggregatibacter aphrophilus: First to be Reported

**DOI:** 10.7759/cureus.97270

**Published:** 2025-11-19

**Authors:** Morihide Kanawa, Naonori Maeda, Munehiro Furuichi, Kazuki Yamazawa, Masayoshi Shinjoh

**Affiliations:** 1 Training and Education, National Hospital Organization (NHO) Tokyo Medical Center, Tokyo, JPN; 2 Pediatrics, National Hospital Organization (NHO) Tokyo Medical Center, Tokyo, JPN; 3 Pediatrics, Keio University School of Medicine, Tokyo, JPN

**Keywords:** aggregatibacter aphrophilus, bacteremia, child, osteomyelitis, spine

## Abstract

Pediatric cases of pyogenic vertebral osteomyelitis are rare; therefore, identifying the causative organism and selecting appropriate antimicrobial therapy can be challenging. In this report, we present the first pediatric case of vertebral osteomyelitis caused by *Aggregatibacter aphrophilus*, identified in an 11-year-old Japanese boy. The patient, who had no history of dental procedures, presented with intermittent fever and lower back pain that persisted for 50 days. *A. aphrophilus* was identified by the 16S rRNA gene sequencing of cultures of intervertebral disc biopsies and repeated blood samples. The patient recovered without complications after six weeks of intravenous antibiotics and two weeks of oral therapy. No surgical intervention was needed. Furthermore, we reviewed this case in the context of 25 previously reported cases identified in the Medline database (1983-2025). This case report indicates that *A. aphrophilus* can cause invasive infection even in previously healthy children. Repeated sampling and 16S rRNA gene analysis can facilitate definitive diagnosis.

## Introduction

The global annual incidence of acute osteomyelitis in pediatrics ranges from 1.2 to 13 cases per 100,000 people, with 10%-25% of cases involving short or non-tubular bones, such as the pelvis, vertebrae, or clavicle [1]. Pediatric pyogenic spondylitis remains extremely rare. Indeed, the reported positivity rates for blood cultures and biopsy cultures are approximately 8% and 40%, respectively [2]. This illustrates the difficulty in identifying the causative organism and selecting an appropriate antimicrobial therapy. *Aggregatibacter aphrophilus* is a facultative anaerobic, gram-negative bacillus, part of the normal oral flora and belongs to the HACEK group (*Haemophilus, Aggregatibacter, Cardiobacterium, Eikenella,* and *Kingella*) [3]. Based on genetic analysis, *Haemophilus aphrophilus* and *Haemophilus paraphrophilus *have been reclassified as *A. aphrophilus *by Nørskov-Lauritsen and Mogens Kilian [4]. *A. aphrophilus* is a causative agent of infective endocarditis, brain abscesses, and osteoarticular infections [5-7]. Owing to its fastidious growth requirements and slow colony development, the isolation of *A. aphrophilus* is often challenging [8], and 16S rRNA gene sequencing is considered a useful tool for its identification. As of August 2025, 25 cases of vertebral osteomyelitis caused by *A. aphrophilus* have been documented. To the best of our knowledge, this is the first reported case of pediatric vertebral osteomyelitis caused by this organism.

This article was previously presented as an oral presentation at the 56th Annual Meeting of the Japanese Society for Pediatric Infectious Diseases on November 16, 2024.

## Case presentation

An 11-year-old Japanese boy presented to our center with intermittent fever and lower back pain that had persisted for 50 days. The patient's growth and development were unremarkable. He had no significant medical history, except for a circumcision performed under general anesthesia nine months prior to presentation, and no history of trauma, dental treatment, or contact with animals.

The patient, who experienced intermittent fever and lower back pain approximately 50 days prior to admission, initially visited a local internal medicine clinic, where he was diagnosed with secondary back pain associated with a common cold and was prescribed antipyretic and analgesic medications. He experienced five febrile episodes prior to admission, with temperatures ranging from 38℃ to 40℃, each lasting from half a day to three days. His back pain showed no improvement, and 27 days prior to admission, he consulted an orthopedic specialist at another hospital. Neurological examinations and plain lumbar spine radiography revealed no abnormalities. As his symptoms persisted, he visited a different internal medicine clinic 19 days prior to admission. Blood tests revealed elevated levels of inflammatory markers, including C-reactive protein (CRP) at 2.87 mg/dL and erythrocyte sedimentation rate (ESR) at 45 mm/h.

However, because there were no signs of clinical urgency, he was prescribed antipyretic and analgesic medications. Fourteen days prior to admission, he revisited the orthopedic department due to worsening pain, which now also involved the left thigh. Neurological examination and lumbar spine radiography findings were unremarkable. Twelve days prior to admission, the patient was referred to our hospital. Lumbar magnetic resonance imaging (MRI), performed to evaluate possible spinal tumors, showed high signal intensity at the posterior-inferior margin of the L4 vertebral body and the superior margin of L5. Although the two sets of blood cultures obtained during the outpatient period for suspected osteomyelitis were negative, the clinical course and imaging findings suggested pyogenic vertebral osteomyelitis, prompting admission for biopsy and treatment.

**Figure 1:**
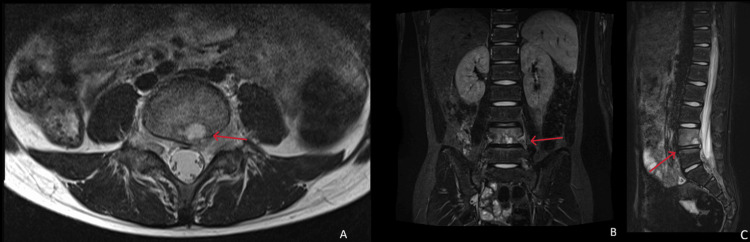
Lumbar spine MRI findings A: T2-weighted axial image shows high signal intensity at the posterior-inferior aspect of the L4 vertebral body (red arrow) B: Short tau inversion recovery (STIR) hyperintensity is observed around the spine (red arrow) C: Sagittal STIR image of the lumbar spine demonstrates high signal intensity at the posterior-inferior endplate of L4 and the superior endplate of L5 (red arrow)

**Figure 2:**
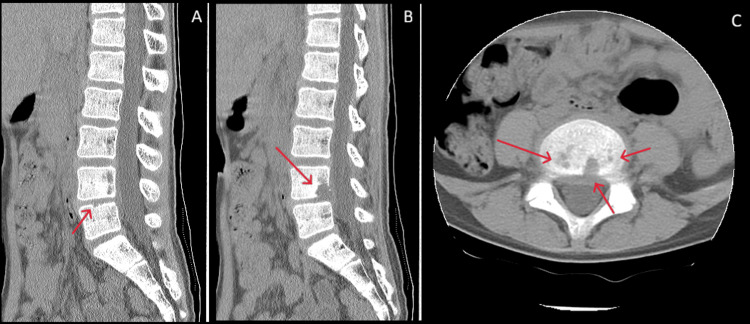
Lumbar spine CT findings A: Irregularity at the superior margin of the L5 vertebral body (red arrow) B: Osteolytic changes with bone destruction in the L4 vertebral body (red arrow) C: Multiple soft tissue densities (red arrows) protruding from the L4/L5 intervertebral disc into the L4 vertebral body

On presentation, the patient’s vital signs and anthropometric measurements were as follows: height, 155.5 cm; weight, 34.95 kg; body temperature, 37.0℃; blood pressure, 98/70 mmHg; heart rate, 101 beats/min; and respiratory rate, 18 breaths/min. He complained of lower back and anterior left thigh pain, both of which worsened with positional changes. No tenderness, guarding, percussion, passive motion, or muscle weakness was observed at the affected site. Neurological examinations revealed no abnormalities. The flip sign was negative, with no signs of intermittent claudication or bladder or bowel dysfunction. The laboratory test results are presented in Table 1. Inflammatory markers were elevated, with a CRP level of 1.57 mg/dL and an ESR of 59 mm/h. Lumbar MRI revealed high signal intensity on T2 star-weighted images at the posterior-inferior margin of the L4 vertebral body and superior margin of L5 (Figure 1). Lumbar computed tomography (CT) revealed multiple soft tissue densities protruding from the L4/5 intervertebral disc into the L4 vertebral body, along with disc bulging, narrowing of the intervertebral disc space, and osteolytic changes with bone destruction at L4 (Figure 2).

**Table 1:** Laboratory data on admission

Parameter	Patient Value	Reference Value
White blood cells	6000/μL	4500-13500/μL
Neutrophils	59.2%	40-70%
Lymphocytes	30.7%	20-50%
Monocytes	9.1%	2-9%
Eosinophils	0.8 %	1-6%
Basophils	0.2%	0-2%
Red blood cell	442×10^4^/μL	435-555×10^4^/μL
Hemoglobin	12.4 g/dL	11.5-16.8 g/dL
Platelet	28.3×10^4^/μL	15.8-34.8×10^4^/μL
Alkaline phosphatase	196 U/L	38-113 U/L
Aspartate aminotransferase	17 U/L	13-30 U/L
Alanine aminotransferase	8 U/L	3-20 U/L
Albumin	4.5 g/dL	4.1-5.1 g/dL
Creatinine	0.53 mg/dL	0.40-0.61 mg/dL
Sodium	139 mmol/L	138-145 mmol/L
Potassium	4.4 mmol/L	3.4-4.7 mmol/L
Chloride	101 mmol/L	98-106 mmol/L
Calcium	9.6 mg/dL	9.4-10.3 mg/dL
C-reactive protein	1.57 mg/dL	<0.14 mg/dL
Procalcitonin	0.182 ng/mL	<0.046 ng/mL
Erythrocyte sedimentation rate	59 mm/hour	2-10 mm/hour
CH50	72.9 U/mL	31.6-57.6 U/mL

**Figure 3:**
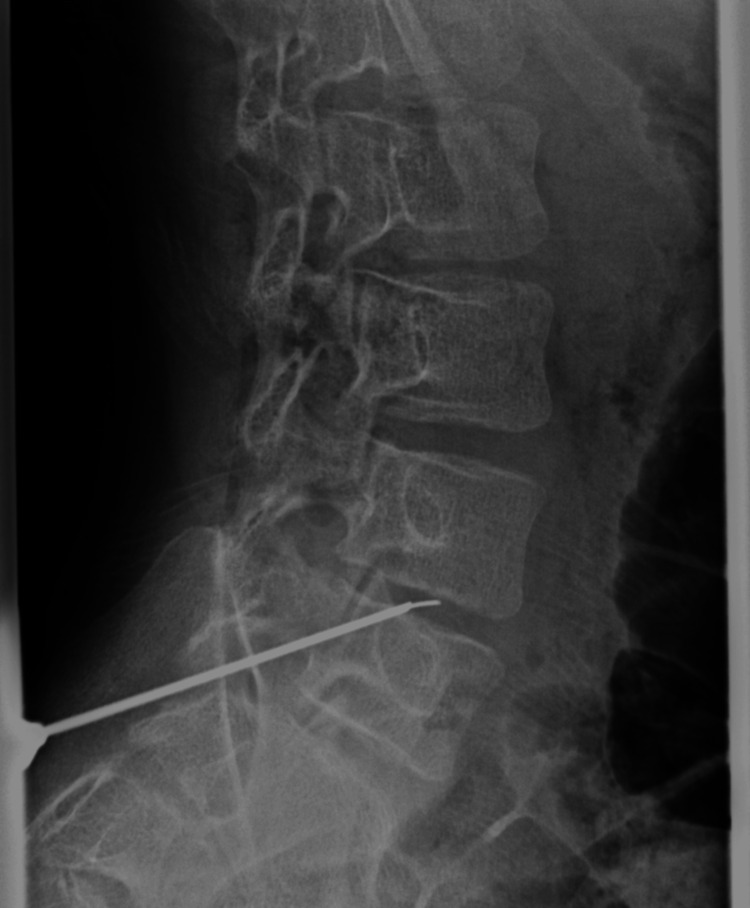
Intraoperative radiograph during percutaneous disc biopsy at the L4/L5 intervertebral disc

On hospital day 1, pyogenic vertebral osteomyelitis was diagnosed based on physical findings, laboratory data, and imaging results. Two sets of blood cultures were obtained, and a percutaneous disc biopsy of the L4/L5 intervertebral disc was performed (Figure 3). No aspirated fluid was obtained; however, the tissue adhering to the biopsy needle was cultured. Empirical antibiotic therapy with cefazolin (150 mg/kg/day) was initiated. 

**Figure 4:**
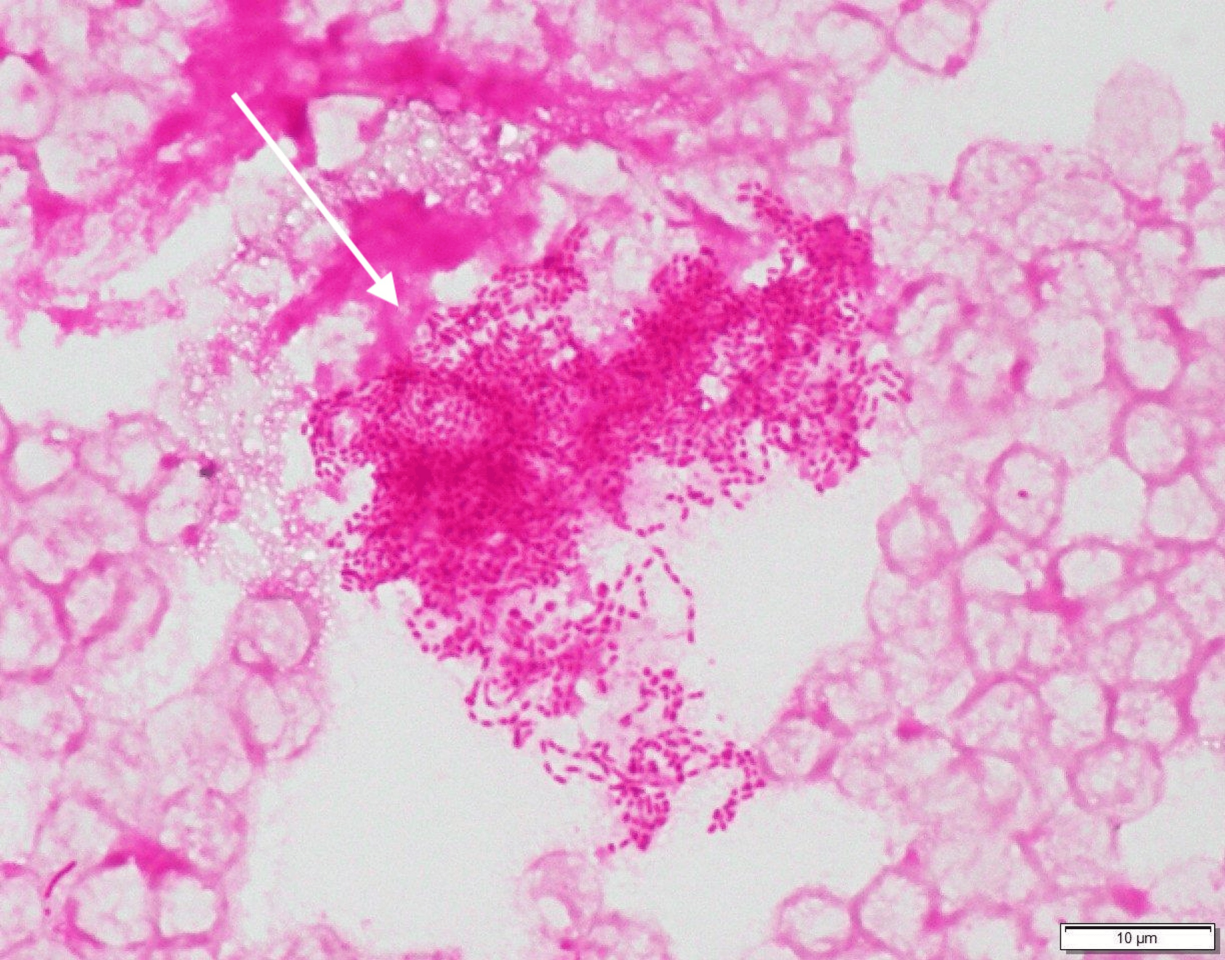
Gram stain of the blood smear showing small gram-negative rods (white arrow).

On hospital day 3, a small number of fastidious gram-negative bacilli grew on chocolate agar from disc biopsy specimens. Based on biochemical characteristics, *A. aphrophilus* was suspected as the causative organism on hospital day 4, and antibiotic therapy was switched to ceftriaxone (4 g/day). On hospital day 6, gram-negative rods were detected in two sets of blood cultures. Gram-negative rods were observed in the smears prepared from the blood specimens (Figure 4).

The organism could not be identified using standard methods at our laboratory; therefore, 16S rRNA gene sequencing was performed at an external reference laboratory, which identified the pathogen as *A. aphrophilus*. Antimicrobial susceptibility testing revealed minimum inhibitory concentrations (MICs) of ≤0.06 μg/mL for cefotaxime, ceftriaxone, levofloxacin, and meropenem; ≤0.12 μg/mL for trimethoprim/sulfamethoxazole; and 0.25 μg/mL for ampicillin, indicating susceptibility to these agents. The MIC for clarithromycin was 32 μg/mL, indicating resistance. The antimicrobial susceptibility results were interpreted with reference to CLSI M45, 3rd edition [9]. Based on these results, the antibiotic regimen was switched to ampicillin at 230 mg/kg/day (8 g/day). Notably, inflammatory markers in blood tests gradually improved over time.

**Figure 5:**
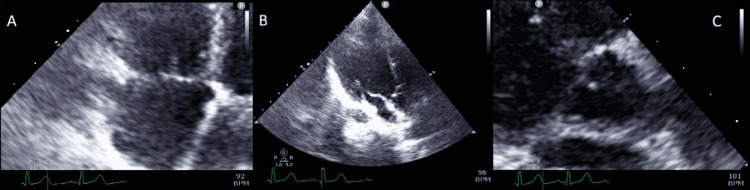
Transthoracic echocardiography demonstrating absence of findings suggestive of infective endocarditis A: Four-chamber view demonstrating evaluation of the tricuspid valve B: Three-chamber view demonstrating assessment of the mitral and aortic valves C: Parasternal view demonstrating visualization of the aortic valve

Blood cultures on hospital days 6 and 9 were incubated for two weeks and showed no growth. Transthoracic echocardiography performed during hospitalization revealed no evidence of vegetation, valvular perforation, aneurysm, or abscess formation. Roth spots were not observed on fundoscopic examination, and there were no findings suggestive of infective endocarditis (Figure 5).

The follow-up lumbar MRI on day 30 and CT on day 44 revealed improvement in the lesions. Intravenous antibiotics were continued for a total of six weeks, after which the patient was discharged with oral amoxicillin 90 mg/kg/day (3 g/day) for an additional two weeks. After completion of antibiotic therapy, the patient remained afebrile, and his back pain resolved.

**Figure 6:**
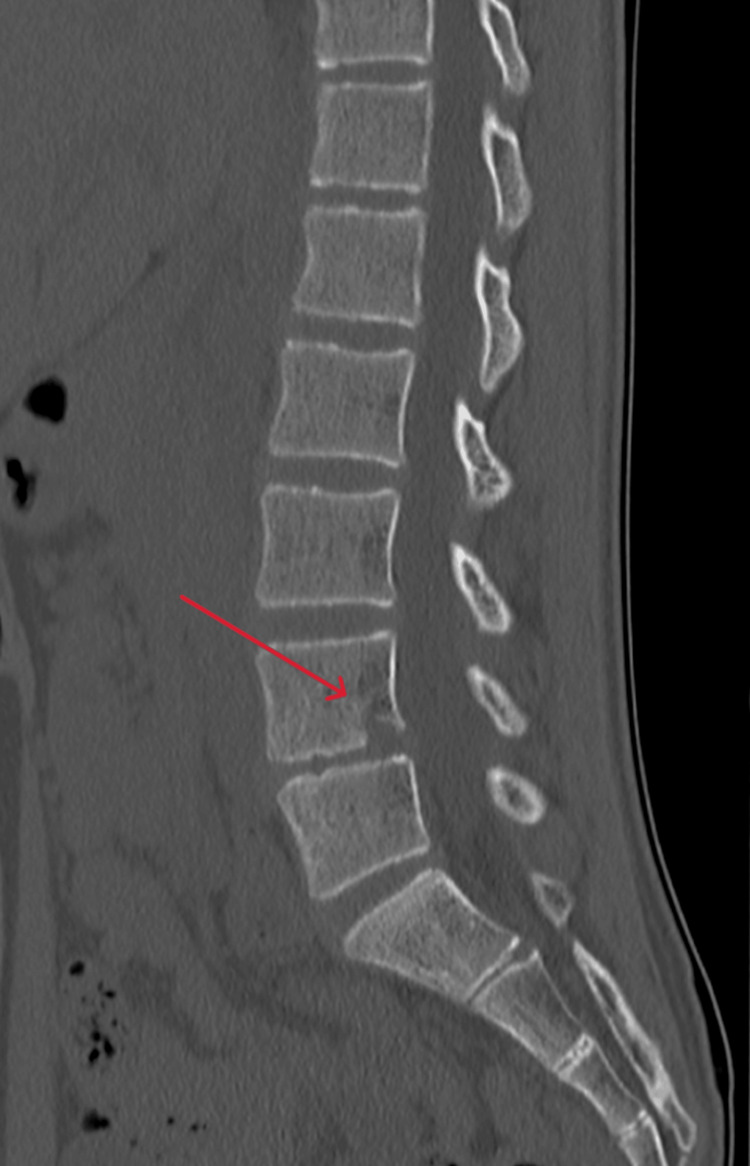
Interval regression of osteolytic changes at the inferior and posterior aspects of the L4 vertebral body on follow-up CT (red arrow)

At the nine-month follow-up, the patient maintained healthy growth in terms of both height and weight. Although narrowing of the L4/L5 intervertebral disc space persisted on plain CT imaging, remodeling progressed over time, and no signs suggestive of recurrent infection were present (Figure 6).

## Discussion

This study presents a pediatric case of pyogenic vertebral osteomyelitis caused by *A. aphrophilus*. The patient, an 11-year-old boy, was successfully treated without recurrence through accurate pathogen identification and the selection of appropriate antibiotics. A literature review was conducted using the Medline (National Library of Medicine) database from December 1983 to August 2025. The following search terms were used: (“*Haemophilus aphrophilus*” OR “Haemophilus paraphrophilus” OR “*Aggregatibacter aphrophilus*”). Additional case reports were identified by reviewing tables and supplementary materials from the retrieved literature. Only 26 cases of pyogenic vertebral osteomyelitis caused by *A. aphrophilus* had been reported as of August 2025, including the present case (Table 2). The median age of the patients was 56 years (range, 11-76 years), and our patient is the youngest reported patient to date. All patients experienced localized pain corresponding to the site of infection, and fever was observed in 20 of the 26 patients. The lumbar spine was the most frequently affected site, reported in 17 of the 26 cases.

**Table 2:** Clinical characteristics of vertebral osteomyelitis caused by A. aphrophilus Abbreviations: AMPC/CVA, amoxicillin and clavulanic acid; AMPC, amoxicillin; ABPC, ampicillin; ABPC/SBT, ampicillin/sulbactam; AZT, aztreonam; CMD, cefamandole; CEZ, cefazolin; CTX, cefotaxime; CTM, cefotiam; CAZ, ceftazidime; CTRX, ceftriaxone; CEX, cephalexin; CP, chloramphenicol; CRFX, ciprofloxacin; CLDM, clindamycin; DOXY, doxycycline; GM, gentamicin; IPM/CS, imipenem-cilastatin; iv, intravenous; LVFX, levofloxacin; NTL, netilmicin; PFLX, pefloxacin; PCG, penicillin G; po, per os; RFP, rifampicin; ST, sulfamethoxazole/trimethoprim

Case no. Author [reference]	Age/sex	Chief complaint	Spinal level	Predisposing factors	Culture	Main antibiotic treatment (duration)	Surgery	Outcome
1. Farrington M, et al. [10]	59/F	Fever, back pain	L2/L3	Hip surgery, epidural catheterization	Drill biopsy specimen, abscess, blood	iv CAZ 3 g/day (11 days) then po RFP 450 mg and DOXY 100 mg/day (3 months)	Yes	Recovery
2. Farrington M, et al. [10]	66/M	Rigors, back pain	L2	No	Open bone biopsy specimen	iv CTX 4 g/day (2 weeks) then po AMPC 2000 mg/day (4 weeks)	Yes	Not mentioned
3. Ho JL, et al. [11]	36/M	Fever, back pain	L2/3	Lip laceration	Needle biopsy specimen, blood	iv PCG 24 million units/day (5 weeks)	No	Recover
4. Petty BG, et al. [12]	69/F	Fever, back pain	L2-4	Lumbar puncture	Abscess	iv CMD (24 days) then CP (46 days)	Yes	Recover
5. Gribble MJ and Hunter T [13]	60/F	Back pain	T11	No	Needle biopsy specimen	iv PCG 18 million units/day (6 weeks)	No	Recover
6. Houssiau FA, et al. [14]	59/M	Fever, back pain	L2	No	Needle biopsy specimen, blood	iv ABPC 8 g/day (3 weeks) then po ABPC	No	Recover
7. Nahass RG, et al. [15]	38/M	Back pain	T11/12	Gingival hemorrhage, dog bite	Vertebral granulation tissue	iv CTRX 2 g/day then antibiotic therapy (4 weeks)	Yes	Recover
8. Stephanian E, et al. [16]	38/M	Fever, neck pain	C5/6	No	Prevertebral and intervertebral granulation tissue	iv penicillin 12 g/day and GM 240 mg/day (2 weeks) then CEZ 6 g/day (4 weeks) then po CEX 2000 mg/day (6 months)	Yes	Recover
9. Brenner KP, et al. [17]	43/M	Rigors, back pain	L3/4	Scratch injury	Vertebral biopsy specimen	iv CRFX 400 mg/day (6 days) then po CRFX 500 mg/day (7 weeks)	Yes	Recover
10. Bejuk D, et al. [18]	40/M	Fever, chest pain	T11/12	No	Blood	CRFX (3 weeks) then AMPC/CVA (5 weeks)	No	Recover
11. Chevalier X and Larget-Piet B [19]	67/M	Fever, back pain	L4/5	No	Discovertebral biopsy specimen	iv PFLX 800 mg/day and NTL (4 weeks) then po PFLX (8 weeks)	No	Recover
12. Wilson CM, et al. [20]	41/M	Fever, abdominal and back pain,	T11	No	Needle biopsy specimen	iv ABPC 8 g/day (4 weeks) then oral antibiotics (3 weeks)	No	Recover
13. O'Driscoll JC, et al. [21]	53/M	Back pain	L4/5	Dental procedure	Blood, abscess	iv CTX 12 g/day then iv CRFX 400 mg/day then po CRFX	Yes	Recover
14. Honan M, et al. [22]	63/M	Back pain	L2/3	No	Blood	iv ABPC and GM (4 weeks) then CTRX (2 weeks)	Not mentioned	Recover
15. Margelli D, et al. [23]	36/M	Fever, back pain	L5/S1	No	Paravertebral tissue	iv CRFX 400 mg/day (6 weeks) then po CRFX 1000 mg/day (4 weeks)	No	Recover
16. Hung CC, et al. [24]	73/F	Fever, back pain, numbness and weakness of the right lower extremity	L4/5	No	Blood	iv CTX 6 g/day (3 weeks) then iv CRFX 600 mg/day (2 weeks) then po CRFX 1000 mg/day (8 weeks)	No	Recover
17. Nagoya H, et al. [25]	73/M	Fever, neck pain, left arm pain	C3/4	Periodontal disease	Needle biopsy specimen	iv CTM 2 g/day (5 weeks and 4 days) then po LVFX 300 mg/day (6 weeks)	Yes	Recover
18. Poullis A, et al. [26]	53/M	Flu-like illness, neck pain	C-spine	Dental procedure	Blood	iv AMPC and CRFX (3 days) then AMPC, CRFX and GM (3 weeks) then po CRFX (2 weeks)	Yes	Recover
19. Colson P, et al. [27]	35/M	Fever, back pain	L4/5	Dental procedure	Abscess	iv CTRX 2 g/day and po CRFX 1500 mg/day (3 weeks) then po CRFX 1500 mg/day (3 weeks)	Yes	Recover
20. Huang ST, et al. [7]	70/F	Neck pain	C6/7	No	Blood	iv CTRX (2 weeks) then AZT (5 weeks)	Yes	Recover
21. Pasqualini L, et al. [28]	63/F	Fever, cervical back pain, weakness of the left upper arm	C6/7	Dental procedure	Blood	iv IPM/CS 1500 mg/day (12 weeks)	No	Recover
22. Chien JT, et al. [29]	68/M	Fever, back pain, delirium	L4/5	Periodontal disease	Blood, abscess	iv AMPC/CVA 3000/600 mg/day and po ST 2400/480 mg/day (4 weeks) then po AMPC/CVA 1750/250 mg/day (8 weeks)	Yes	Recover
23. Bernard F, et al. [30]	76/M	Fever, back pain	L3/4	Periodontal disease	Abscess	iv CTRX 4 g/day (6 weeks)	No	Recover
24. Sogi M, et al. [31]	46/M	Weakness and numbness in both legs, leg pain	L4/5	Periodontal disease	Abscess	iv ABPC/SBT 12g/day (132 days) then po CLDM	Yes	Recover
25. Khurana A, et al. [32]	44/M	Fever, back and flank pain	L4/5	Right hip fracture, IVC filter	Abscess	iv CTRX (6 weeks)	Yes	Recover
26. Kanawa M et al. (Present case)	11/M	Fever, back pain, leg pain	L4/5	Circumcision	Blood, needle biopsy specimen	iv ABPC 300 mg/kg/day (5 weeks) then po AMPC 90 mg/kg/day (2 weeks)	No	Recover

Although *A. aphrophilus* causes infective endocarditis and brain abscesses, no reports have described concurrent vertebral osteomyelitis with either of these conditions.

The primary sources of osteomyelitis caused by *A. aphrophilus* are dental procedures, oral lesions, and contact with animals. Huang et al. reported that 39% of the patients with invasive infections caused by *A. aphrophilus* had a history of dental treatment [7], while Maraki et al. suggested that contact with dogs may be a potential source of infection [33]. Among the 26 reported cases of vertebral osteomyelitis, 10 cases had a history of dental procedures or oral lesions, and two cases had a history of animal bites or injuries. However, in our case, our patient had no history of dental treatment, oral disease, cardiac disease, trauma, or contact with animals, which are common pathways for bacterial entry. Although the patient had undergone circumcision under general anesthesia nine months prior to admission, its relevance to the current infection remains unclear.

As osteoarticular infections often require prolonged treatment, it is necessary to identify the causative agent; however, the pathogen remains unidentified in approximately 33%-55% of such infections [34]. *A. aphrophilus*, the causative organism in this case, has poor biochemical reactivity, fastidious growth requirements, and slow colony formation, contributing to the low number of reported cases [8]. Identifying the pathogen in pyogenic vertebral osteomyelitis remains challenging, with reported positivity rates of only 8% for blood cultures and 40% for biopsy cultures [2]. In this context, 16S rRNA gene sequencing is a valuable diagnostic tool, and future accumulation of such cases is anticipated. Although advances in imaging techniques have facilitated the earlier diagnosis of vertebral infections, timely and appropriate antimicrobial treatment relies on microbiological confirmation. Therefore, it is essential to actively perform culture testing, including percutaneous biopsy, and employ appropriate genetic methods, such as 16S rRNA gene sequencing, when identification of the isolated organism is difficult.

Furthermore, our review of 26 cases of pyogenic vertebral osteomyelitis caused by *A. aphrophilus* revealed that blood cultures failed to detect the organism in 14 cases, whereas percutaneous biopsy specimens yielded positive results in 20 of the 21 cases. In the present case, both sets of blood cultures obtained before admission were negative; however, the pathogen was ultimately identified by cultures obtained during an extended untreated period, which allowed for microbial detection. These findings underscore the importance of repeated blood cultures and obtaining biopsy specimens whenever possible to increase the likelihood of pathogen identification.

Following the antimicrobial susceptibility testing, the primary antibiotics administered were ciprofloxacin, ceftriaxone, and ampicillin. Excluding cases in which the treatment duration was not reported, the median duration of intravenous antibiotic therapy was five weeks (range: 6-18 weeks and 6 days), followed by a median duration of three weeks (range: 0-24 weeks) of oral antibiotic therapy. Surgical intervention was required in 14 of the 25 patients. All patients had favorable outcomes. Guidelines from the American Heart Association recommend ceftriaxone as a reasonable treatment option for infective endocarditis caused by HACEK [34]. According to the clinical practice guidelines of the Pediatric Infectious Diseases Society and the Infectious Diseases Society of America, a minimum of three weeks of antibiotic therapy is recommended for pediatric osteomyelitis [1]. In cases of pyogenic vertebral osteomyelitis, delayed treatment may lead to irreversible complications, including spinal deformity and neurological deficits.

As this review is based solely on published case reports, publication bias cannot be excluded, and milder or undiagnosed cases may have been underreported. Future multicenter accumulation of microbiologically confirmed cases will help clarify the clinical spectrum of *A. aphrophilus* vertebral infections.

## Conclusions

In summary, we report the first pediatric case of pyogenic vertebral osteomyelitis caused by *A. aphrophilus*. This case highlights that *A. aphrophilus* can be a causative pathogen even in previously healthy children without a history of oral procedures and should therefore be considered in the differential diagnosis of vertebral infections. Although reports of *A. aphrophilus*-related vertebral osteomyelitis remain limited, repeated blood cultures, tissue cultures whenever possible, and 16S rRNA gene sequencing may contribute to increased detection and accumulation of cases, ultimately enhancing our understanding of the disease.
